# Investigation of drug-related problems in patients hospitalized in chest disease wards: A randomized controlled trial

**DOI:** 10.3389/fphar.2022.1049289

**Published:** 2023-01-10

**Authors:** Muhammed Yunus Bektay, Mesut Sancar, Fatmanur Okyaltirik, Bulent Durdu, Fikret Vehbi Izzettin

**Affiliations:** ^1^ Clinical Pharmacy Department, Faculty of Pharmacy, Bezmialem University, Istanbul, Turkey; ^2^ Clinical Pharmacy Department, Health Science Institute, Marmara University, Istanbul, Turkey; ^3^ Clinical Pharmacy Department, Faculty of Pharmacy, Marmara University, Istanbul, Turkey; ^4^ Department of Chest Diseases, Medical Faculty, Bezmialem Vakif University, Istanbul, Turkey; ^5^ Department of Infectious Diseases and Clinical Microbiology, Medical Faculty, Bezmialem Vakif University, Istanbul, Turkey

**Keywords:** clinical pharmacist, drug-related problems, chest diseases, cognitive pharmacy services, pharmaceutical care

## Abstract

**Objective:** According to the World Health Organization (WHO), chest diseases are among the 10 diseases that cause the highest mortality worldwide. Drug-related problems (DRPs), readmission, and antimicrobial resistance are critical problems in chest disease wards. Active involvement of clinical pharmacists (CPs) who are focused on reducing the risks of potential problems is needed. The aim of this study is to investigate the effects of pharmaceutical care (PC) services on the pulmonology service.

**Method:** A randomized controlled trial at a university hospital in Istanbul was conducted between June 2020 and December 2021. The participants were randomized into the control group (CG) and intervention group (IG). In the CG, CPs identified and classified the DRPs according to Pharmaceutical Care Network Europe v9.0 (PCNE) and provided solutions to DRPs for the IG. The effect of PC services was evaluated by the number and classification of DRPs, and readmissions within 30 days were compared between the two groups.

**Results:** Out of 168 patients, 82 were assigned to the IG. The average number of medicines administered per patient in the CG and IG was 14.45 ± 7.59 and 15.5 ± 6.18, respectively. In the CG and IG, the numbers of patients with DRPs were 62 and 46, respectively. The total number of DRPs was 160 for CG and 76 for IG. A statistically significant difference was found in favor of the IG, in terms of the number of patients with DRPs, the total number of DRPs, and readmission within 30 days (*p* < 0.05).

**Conclusion:** In this study, CP recommendations were highly accepted by the healthcare team. Pharmaceutical care services provided by CPs would decrease possible DRPs and led to positive therapeutic outcomes. Cognitive clinical pharmacy services have beneficial effects on health care, and these services should be expanded in all settings where patients and pharmacists are present.

## Introduction

According to data from the World Health Organization (WHO), chest diseases are among the 10 diseases that cause the highest mortality worldwide. Diseases such as chronic obstructive pulmonary disease (COPD), lower respiratory tract infections, and lung and related tissue cancers constitute three of the 10 diseases that cause the most death (World Health Organization, 2019a). According to the WHO (World Health Organization, 2019b), mortality per 100,000 people in Turkey due to trachea, bronchial, or lung cancer is 42.05; COPD-related mortality is 30.08; and lower respiratory tract infection-related deaths are 15.4. Globally, lower respiratory tract infections cause up to 4 million deaths annually. Lower respiratory tract infections are a common problem and affect children more than adults, especially in low- and middle-income countries (Lozano et al., 2012). The treatment of lower respiratory tract infections, which are often caused by bacterial and viral pathogens, becomes more difficult due to antimicrobial resistance. In the last decade, viral infections such as SARS, MERS, and COVID-19 have caused pandemics and deaths worldwide (ECDC PEC for DC, 2021). The increased prevalence of pulmonary infections, obstructive lung diseases, and cancer cases requires intense interest. The treatment of these diseases requires multidrug therapies and a multidisciplinary healthcare team. About one-third of US people have drug therapy difficulties, which are especially prevalent in patients undergoing treatment for several concomitant medical disorders (Maher et al., 2014). Drug-related problems (DRPs) are common in patients with chest diseases such as pneumonia, COPD, asthma, and lung cancer (Ottenbros et al., 2014; Apikoglu-Rabus et al., 2016; Guillamet et al., 2016).

Clinical pharmacy services consist of patient-oriented pharmacy services in all healthcare settings. A clinical pharmacist (CP) who works on reducing the risks of pulmonary diseases should provide the necessary services using the theoretical and clinical training they received in order to optimize health-related outcomes (American College of Clinical Pharmacy ACCP, 2008; Kara et al., 2021). Drug-related problems are common in chest diseases, and the nature of these DRPs is usually quite complex. The high number of comorbidities in elderly patients increases the likelihood of DRPs and may worsen the prognosis. DRPs require caution in prescribing, dispensing, and administering drugs. In addition, DRPs may increase readmission rates. The DRPs constitute a significant burden for health systems and patients alike (Guillamet et al., 2016; Pharmaceutical Care Network Europe Association, 2020).

There are several different approaches to the prevention of DRPs. Physicians, CPs, and other healthcare professionals play an important role in reducing and preventing the DRPs, mortality, and morbidity. In addition to patient-oriented clinical pharmacy services such as medication reviews, patient education and medication reconciliation can offer solutions to possible problems that may arise during prescribing. Irrational drug use, comorbidities, readmission, adverse events, and drug interactions could be counted as the most common DRPs among hospitalized patients (Rankin et al., 2018).

### Aim of the study

The aim of this study is to detect, classify and resolve the DRPs in the chest disease ward.

## Materials and methods

### Study design and sample size

A randomized controlled study conducted on patients who admitted to pulmonology service at a tertiary-care university hospital in Istanbul, Turkey between June 30th, 2020, and December 31st, 2021. The patients with COVID-19 infection and in need of intensive care were excluded. Patients diagnosed with any of the ICD code of J05, J9-22, J40-47, J69, J85–J86 were included in the study (Table 1).

**TABLE 1:** List of ICD-10 Codes included in the study.

***J05***	Acute obstructive laryngitis [croup] and epiglottitis
***J09***	Influenza due to identified zoonotic or pandemic influenza virus
***J10***	Influenza due to identified seasonal influenza virus
***J11***	Influenza, virus not identified
***J12***	Viral pneumonia, not elsewhere classified
***J13***	Pneumonia due to Streptococcus pneumoniae
***J14***	Pneumonia due to Haemophilus influenzae
***J15***	Bacterial pneumonia, not elsewhere classified
***J16***	Pneumonia due to other infectious organisms, not elsewhere classified
***J17***	Pneumonia in diseases classified elsewhere
***J18***	Pneumonia, organism unspecified
***J20***	Acute bronchitis
***J21***	Acute bronchiolitis
***J22***	Unspecified acute lower respiratory infection
***J40***	Bronchitis, not specified as acute or chronic
***J41***	Simple and mucopurulent chronic bronchitis
***J42***	Unspecified chronic bronchitis
***J43***	Emphysema
***J44***	Other chronic obstructive pulmonary disease
***J45***	Asthma
***J46***	Status asthmaticus
***J47***	Bronchiectasis
***J69***	Pneumonitis due to solids and liquids
***J85***	Abscess of lung and mediastinum
***J86***	Pyothorax

The study subjects were selected based on the inclusion criteria (confirmed diagnosis of any of these ICD codes: J05, J9-22, J40-47, J69, and J85–J86) and informed consent. The patients were randomized using an algorithm created by software called Research Randomizer^®^. Randomization was completed over the numbers assigned to the patients to be included in the control group (CG) and intervention group (IG).

In accordance to published literature, we estimated the minimum sample size a sample size of 85 was required within a 5% margin of error and confidence intervals (CI) of 95%. According to our sample size calculations, the effect size (d) was calculated as 0.5540, with Alpha (α) 0.05, Beta (β) 0.95, at least 75 patients should be included for each group, and the power of the study was calculated as 0.9510.

### Data collection

The patients' demographic factors (age, gender, body weight), comorbid diseases, prescribed medicine (dosing, frequency, and treatment duration) were recorded. In addition, blood pressure, heart rate, oxygen saturation, respiratory rate, and laboratory findings (e.g., creatinine, uric acid, fasting blood glucose, hemogram, LACE index scores) on admission were recorded (van Walraven et al., 2010). Meanwhile, the number of prescribed medicine or over-the-counter medications were collected. The identification of DRPs made by the pharmacists according to recent guidelines, UpToDate® clinical decision support system and evidence-based medicine.

**FIGURE 1:**
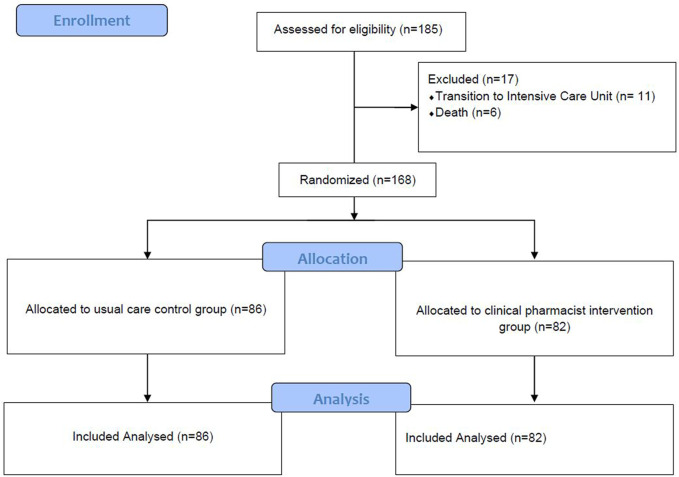
Study flowchart.

The assessment of clinical significance was performed by experienced clinical pharmacists. The DRPs and clinical significance were evaluated using the PCNE v9.0 drug related problems classification system. This study has been reported according to recommendation of Consolidated Standards of Reporting Trials (CONSORT) standards and study flow chart given in Figure 1.

### Clinical pharmacist intervention

The drugs used by the patients within 24–48 h after hospitalization were recorded. In the case of new prescribing, the recommendations made by the clinical pharmacist to the physician are recorded in intervention group. The medication reconciliation report evaluated before the discharge of the patients and possible omissions discussed with the physician. A detailed medication reviews accomplished by the clinical pharmacist during hospitalization of the participants.

The CP attended routine daily patient visits and offered solutions to DRPs. Interventions for DRPs were carried out face-to-face, during the routine daily patient visits and verbal recommendations made to the physician in charge. The CP's interventions consisted of a verbal advice to avoid or address potential DRPs at any level of the medication prescribing cascades. As a result, through the CP's daily medication reviews, intervention recommendations such as adding or stopping medication, recommending an alternative treatment, changing the route of administration, preventing, or managing side effects, optimizing drug administration route, or adjusting dosage were provided by CP during hospital stay of the patients. In the evaluation of the probable drug-drug interactions, only “Major/D” and “Contraindicated/X” levels interactions were considered as pDDIs. Contraindicated interactions require the avoidance of medication combinations, whilst major/D level interactions refer to treatment modification taking into consideration. For information on pDDIs, dose, and medication administrations drug monographs, Uptodate Drug Information, Micromedex Drug Information, and other resources were utilized. DRPs were classified using the PCNE V9.0 as classification method (Ayhan et al., 2022).

Drug-related problems (DRPs) were identified using the Turkish version of the PCNE Classification scheme for Drug-Related Problems v9.0. which was validated by Pharmaceutical Care Network Europe Association working group. (https://www.pcne.org/upload/files/352_PCNEV9.0_Turkish.pdf). The basic classification now has 3 primary domains for problems, 9 primary domains for causes and 5 primary domains for Planned Interventions, 3 primary domains for level of acceptance (of interventions) and 4 primary domains for the Status of the problem. However, on a more detailed level there are 7 grouped sub domains for problems, 43 grouped subdomains for causes and 17 grouped sub domains for interventions, and 10 subdomains for intervention acceptance. Those sub-domains can be seen as explanatory for the principal domains.

The data collected for DRPs were demographics, number of prescribed medicines, duration of hospital stay, the type and causes of the problem, interventions provided to resolve the problem by the clinical pharmacist, and the outcomes of the interventions. Verbal recommendations were accomplished by the clinical pharmacist and discussed with the physicians, and the acceptance/rejection rate was recorded.

The rate of 30-day readmission after discharge was recorded. LACE index scores were used to assess whether the control and intervention groups had similar characteristics in terms of readmission (van Walraven et al., 2010).

These interventions were made in the form of post-prescription evaluation, review, and reporting. Possible and real drug-related problems that may arise according to the specific characteristics of the drugs and possible unsuitable drugs were identified, and solutions were conveyed to the responsible physician.

### Main outcome measure

The main outcome measures were number, rate of intervention made by the clinical pharmacist, acceptance rate of suggested intervention, length of hospital stay, and 30-day readmission.

### Statistical analysis

As descriptive statistics, mean, median, standard deviation, and interquartile range [IQR] or count and percentages are given for continuous variables. The frequency and percentage are given for categorical variables. The normality of continuous variables was tested using the Kolmogorov–Smirnov test. The difference among groups was analyzed using the independent t-test or Mann–Whitney U test. Chi-squared tests are used to investigate the relationship between categorical variables. The missing data were excluded from the analysis. All the data were analyzed using SPSS version 26^®^ and Jamovi version 1.6.

## Results

### Demographics, medications, and drug-related problems

Out of the patients included in our study, 86 of 168 patients were randomly assigned to the CG and 82 patients to the IG. The number of males was 58 (67.44%) in the CG and 39 (47.56%) in the IG. The mean age of the patients of the CG and IG was calculated as 72.19 ± 12.73 and 68.80 ± 16.30, respectively.

Most of the patients included in our study had at least one concomitant disease in addition to chest diseases. The mean number of diseases per patient in the CG and IG was recorded as 3.71 ± 1.74 and 3.66 ± 1.67, respectively. More than 70% of the patients of the CG appeared to have three or more comorbidities. On the other hand, 59 (71.95%) patients in the intervention group had three or more comorbidities.

Pneumonia was the leading cause of the hospitalization within our sample size. About two-thirds 57, (66.28%) of the patients in the CG required hospitalization due to pneumonia. The number of patients with hypertension (HT) was recorded in 42 (48.84%) and HT was the most recorded comorbid disease in the CG. The number of patients with coronary artery disease (CAD) was recorded with 35, (40.70%) patients (Table 2). In intervention group the most common reason of hospital stay was pneumonia and the number of patients who admitted to chest disease ward was 57, (69.51%) patients (Table 2). The leading comorbidities for IG were hypertension and COPD 49, (59.75%), and 32, (39.02%) of the patients respectively. The mean number of prescribed drugs during hospital stay per patient was recorded as 14.45 ± 7.59 and 15.5 ± 6.18 in the CG and IG, respectively (Table 2).

**TABLE 2:** Patient Characteristics.

	Control Group (n = 86)	Intervention Group (n = 82)	*p*
Sex n, (%)			>0.05
Female	28, (32.56%)	43, (52.44%)	
Male	58, (67.44%)	39, (47.56%)	
BMI (Median [IQR])	25.95, [23.43–28.07]	25.20, [23.40–27.70]	>0.05
Weight (kg)	75, [70–80]	71, [65–80]	
Height (m)	1.7, [1.66–1.74]	1.67, [1.6–1.75]	
Age (mean ± SD)	72.19 ± 12.73	68.80 ± 16.30	>0.05
No of Comorbidities (mean ± SD)	3.71 ± 1.74	3.66 ± 1.67	>0.05
1 (n, %)	6, (6.98%)	9, (10.97%)	
2 (n, %)	18, (20.93%)	14, (17.07%)	
3 (n, %)	22, (25.58%)	16, (19.51%)	
4 (n, %)	11, (12.79%)	17, (20.73%)	
5 (n, %)	16, (18.60%)	12, (14.63%)	
6 (n, %)	6, (6.98%)	11, (13.41%)	
7 (n, %)	5, (5.81%)	3, (3.65%)	
8 (n, %)	2, (2.33%)	–	
Most common 10 Comorbidities (n, %)			N.A.
Hypertension	42, (48.84%)	49, (59.75%)	
Coronary Artery Disease	35, (40.70%)	27, (32.93%)	
Chronic obstructive pulmonary disease	31, (36.05%)	32, (39.02%)	
Type 2 Diabetes Mellitus	27, (31.40%)	21, (25.61%)	
Pulmonary Embolism	24, (27.91%)	13, (15.85%)	
Cancer	22, (25.58%)	14, (17.07%)	
Asthma	12, (13.95%)	11, (13.41%)	
Atrial Fibrillation	12, (13.95%)	4, (7.87%)	
Kidney failure	12, (13.95%)	14, (17.07%)	
Length of Hospital Stay (day) (mean ± SD)	11.57 ± 8.56	9.72 ± 6.08	*p* > 0.05
<10 (n, %)	44, (51.16%)	51, (62.20%)	
10-19 (n, %)	28, (32.56%)	26, (31.71%)	
20-30 (n, %)	9, (10.46%)	4, (4.88%)	
>30 (n, %)	5, (5.82%)	1, (1.12%)	
Number of Drugs per patient (mean ± SD)	14.45 ± 7.59	15.5 ± 6.18	*p* > 0.05
Charlson Comorbidity Index (CCI) (Median, [IQR])	5, [3–7]	4, [3–6]	*p* > 0.05

**FIGURE 2:**
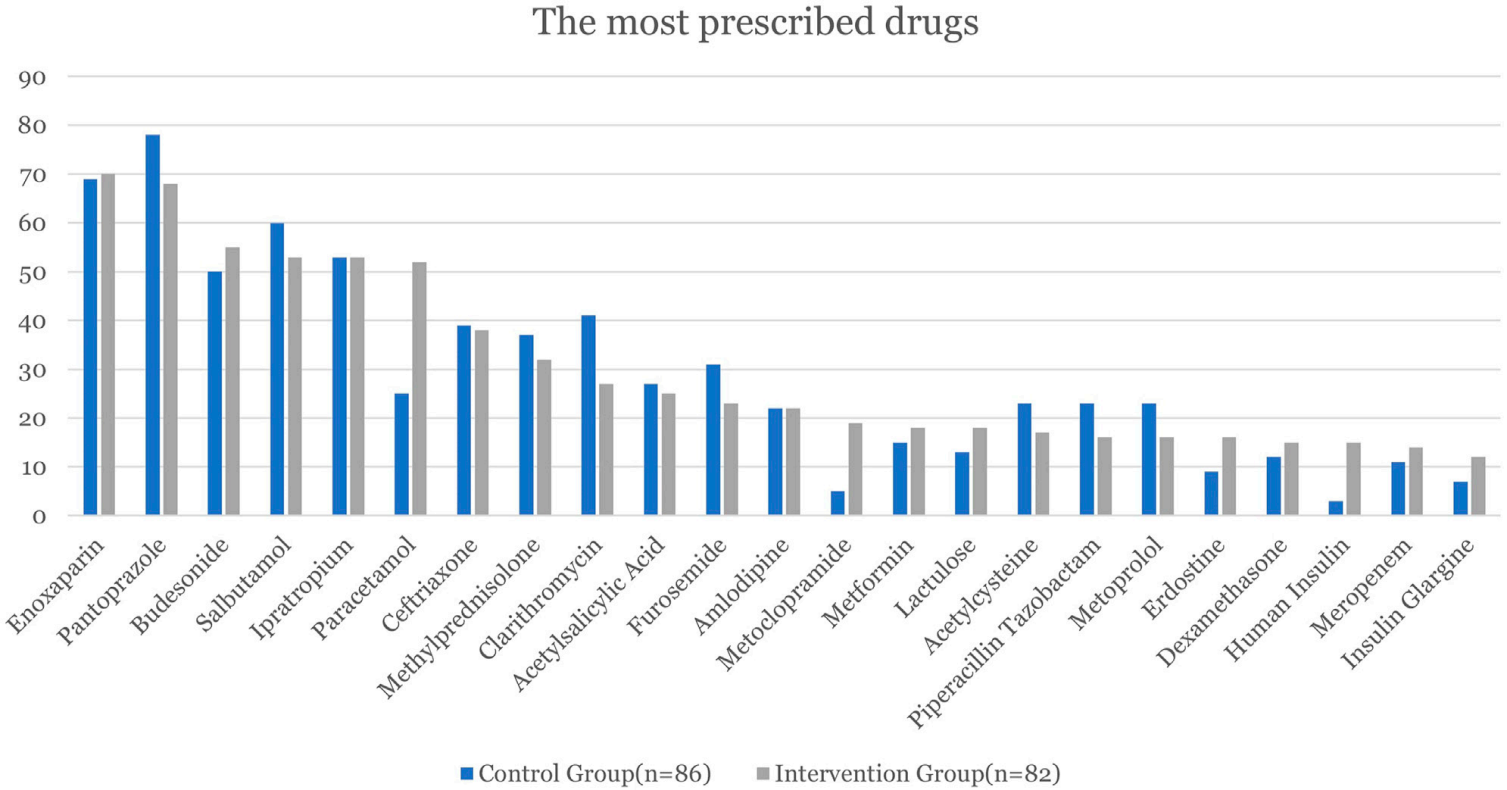
Most used drugs and the number and percentage of patients using them.

The most frequently used drugs in the CG were recorded as pantoprazole, enoxaparin, and salbutamol, 78 (90.7%), 69 (80.23%), and 60 (69.77%) patients respectively. On the other hand, the most frequently used drugs in the CG were recorded as enoxaparin, pantoprazole, and budesonide, 70 (85.37%), 68 (82.93%), and 55 (67.07%) patients respectively (Figure 2).

According to our findings the mean length of stay of patients allocated into CG and IG were 11.57 ± 8.56 and 9.72 ± 6.08 days respectively given in Table 2.

Although there was a nominal difference in the analyses made on the hospital stay, this was not found to be statistically significant (*p* > 0.05). Many patients in both the CG and IG were discharged within 10 days, and the mean length of stay was 11.57 ± 8.56 and 9.72 ± 6.08, respectively.

In our study, 44 (51.16%) of 86 patients in the CG need hospital admission within 30 days after discharge. On the other hand, the number of the rate of 30-day readmission for IG was observed as 18 (21.95%) and a statistically significant difference were obtained (*p* = 0.006) given in Table 3.

**TABLE 3:** Drug-related problems identified in the control and intervention groups.

	Control group (n = 86)	Intervention group (n = 82)	*p*
Number of patients with DRPs, n (%)	62 (72.10%)	46 (56.10%)	<0.05^a^
Total no. of DRPs (n)	160	76	0.006^b^
DRPs per patient (mean ± SD)	2.58 ± 2.18	1.63 ± 1.29	<0.05b
Accepted interventions, n (%)	-	64 (84.21%)	-
Unaccepted interventions, n (%)	-	14 (12.42%)	-
LACE Index for Readmission (mean ± SD)	11.33 ± 3.07	12.2 ± 2.38	*p* > 0.05^a^
Readmissions in 30 days (n, %)	44 (51.16%)	18 (21.95%)	0.006^a^

^a^ Pearson chi-squared test.

^b^ Mann–Whitney U test.

### Drug-related problems and pharmacist interventions

In our study, DRPs were assessed according to PCNE v9.0. No intervention was made by the clinical pharmacist for the CG. Possible and existing DRPs in the control group were identified and recorded. The assessed DRPs, recommendations by the clinical pharmacist and acceptance rates for the CG and IG are given in Tables 3, 4

**TABLE 4:** Main causes of DRPs according to PCNE v9.0.

The basic classification	Control Group (n = 86)		Intervention Group (n = 82)	
	Total	%	Total	%
Problems (also potential)	160		76	
P1 Treatment effectiveness	42	26.25	37	48.68
P1.1 No effect of drug treatment despite correct use	3	1.88	2	2.63
P1.2 Effect of drug treatment not optimal	23	14.38	30	40.00
P1.3 Untreated symptoms or indication	-	-	5	6.67
P2. Treatment safety	118	73.75	37	48.68
P2.1 Adverse drug event (possibly) occurring	118	73.75	37	48.68
P3. Other	-	-	2	2.63
P3.2 Unclear problem/complaint. Further clarification necessary (please use as escape only)	-	-	2	2.63
Causes (including possible causes for potential problems)	160		79	
C1. Drug selection	131	81.88	39	49.37
C1.1 Inappropriate drug according to guidelines/formulary	27	16.88	19	24.05
C1.2 No indication for drug	0	0	1	1.27
C1.3 Inappropriate combination of drugs, or drugs and herbal medications, or drugs and dietary supplements	82	51.25	4	5.06
C1.4 Inappropriate duplication of therapeutic group or active ingredient	-	-	1	1.27
C1.5 No or incomplete drug treatment in spite of existing indication	21	13.13	7	8.86
C1.6 Too many different drugs/active ingredients prescribed for indication	-	-	7	8.86
C2 Drug form	-	-	1	1.27
C2.1 Inappropriate drug form/formulation (for this patient)	-	-	1	1.27
C3 Dose selection	29	18.13	32	40.51
C3.1 Drug dose too low	8	5.00	17	21.52
C3.2 Drug dose of a single active ingredient too high	19	11.88	11	13.92
C3.3 Dosage regimen not frequent enough	1	0.63	1	1.27
C3.4 Dosage regimen too frequent	1	0,63	2	2.53
C3.5 Dose timing instructions wrong, unclear or missing	-	-	1	1.27
C4 Treatment duration	58	36.25	-	-
C5 Dispensing	-	-	-	-
C6 Drug use process	-	-	3	3.80
C6.1 Inappropriate timing of administration or dosing intervals by a health professional	-	-	2	2.53
C6.4 Drug not administered at all by a health professional	-	-	1	1.27
C7 Patient related	-	-	2	2.53
C7.8 Patient unintentionally administers/uses the drug in a wrong way	-	-	1	1.27
C7.10 Patient unable to understand instructions properly	-	-	1	1.27
C8 Patient transfer related	-	-	-	-
C9. Other	-	-	2	2.53
C9.3 No obvious cause	-	-	2	2.53
Planned Interventions	160		76	
I0. No intervention	160	100	5	6.58
I1. At prescriber level	-	-	67	88.16
I1.1 Prescriber informed only	-	-	4	5.26
I1.3 Intervention proposed to prescriber	-	-	6	7.89
I1.4 Intervention discussed with prescriber	-	-	57	75.0
I2. At patient level	-	-	2	2.63
I2.1 Patient (drug) counselling	-	-	2	2.63
I3. At drug level	-	-	1	1.32
I3.2 Dosage changed to	-	-	1	1.32
I4. Other	-	-	1	1.32
I4.1 Other intervention (specify)	-	-	1	1.32
Intervention Acceptance	160		76	
A1. Intervention accepted	-	-	64	84.21
A1.1 Intervention accepted and fully implemented	-	-	53	69.74
A1.2 Intervention accepted, partially implemented	-	-	5	6.58
A1.3 Intervention accepted but not implemented	-	-	5	6.58
A1.4 Intervention accepted, implementation unknown	-	-	1	1.32
A2. Intervention not accepted	-	-	7	9.21
A2.2 Intervention not accepted: no agreement	-	-	4	5.26
A2.3 Intervention not accepted: other reason (specify)	-	-	1	1.32
A2.4 Intervention not accepted: unknown reason	-	-	2	2.63
A3. Other	160	100	5	6.58
A3.2 Intervention not proposed	160	100	5	6.58
Status of the DRP	160		76	
O0. Problem status unknown	-	-	3	3.95
O1. Problem solved	-	-	60	78.95
O2. Problem partially solved	-	-	7	9.21
O3. Problem not solved	160	100	6	7.89
O3.1 Problem not solved, lack of cooperation of patient	-	-	3	3.94
O3.2 Problem not solved, lack of cooperation of prescriber	-	-	3	3.94

.

As a result, according to the PCNE classification in the CG, DRPs detected in 62 (72.1%) patients. The total number of DRPs identified by the clinical pharmacist is 160 for CG. For the intervention groups clinical pharmacist made a total of 76 recommendations to 46 (56.1%) patients (Tables 3, 4). A statistically significant difference was observed in the dichotomous analysis of the presence of DRPs detected in the control and intervention groups (χ2: 5.379; *p* < 0.05). According to the results of the statistical analysis using the Mann-Whitney U test on the number of DRPs seen in the patients, a significant difference was found between the CG and IG (U = 2699; z = −2.751; *p* = 0.006) (Table 3).

While 42 (26.25%) of the DRPs detected in the CG were related with treatment effectiveness, and 118 (73.75%) were associated with treatment safety given in table 3. Among the DRPs detected in the control group, the most common cause was drug selection with 131 (81.88%) times. On the other hand, the treatment duration was the second most common cause of drug-related problem in CG with 58 (36.25%) (Table 4). In intervention group 37 (48.68%) of the DRPs were related with treatment effectiveness and 37 (48.68%) were associated with treatment safety. The most common cause of DRPs in the intervention group is drug selection 39 (49.37%) and dose selection 32 (40.51%) constitutes the second most common problem. It was recorded that the vast majority of interventions suggested by the clinical pharmacist to IG were at the prescriber level 67 (88.16%). The number of interventions accepted by physician was 64 (84.21%) (Table 4) and the examples of interventions of CP given in Table 5.

**TABLE 5:** Examples of drug-related problems identified in the control and intervention groups and recommended by the pharmacist.

The basic classification	Examples of Drug-Related Problems
Problems (also potential)	
P1 Treatment effectiveness	Recommendation for change due to increased CRP in a patient using levofloxacin.
P2. Treatment safety	Recommendation due to metoclopramide drug-drug interaction.
P3. Other	Suggestion of an alternative dosage form for the drug to be the appropriate dosage form.
Causes (including possible causes for potential problems)	
C1. Drug selection	Modification recommendation due to clarithromycin-Silodosin drug-drug interaction.
C2 Drug form	Dosage form recommendation in the patient used in pediatric dosage form.
C3 Dose selection	Ampicillin-Sulbactam dosing recommendation.
C4 Treatment duration	-
C5 Dispensing	-
C6 Drug use process	Suggestion for the patient in the lactation period to express and store milk before drug use.
C7 Patient related	Concurrent unnecessary antibiotic use was detected in the patient who received outpatient antibiotic therapy. Suggestion to organize antibiotic therapy
C8 Patient transfer related	-
C9. Other	Although there are two different drugs from the same group in the drug order, one of them is not used.
Planned Interventions	
I0. No intervention	Somnolence assessment recommendation in a patient using clonazepam.
I1. At prescriber level	Prescription modification recommendation due to irbesartan duplication
I2. At patient level	Suggestion to breast-feed within 4 hours after taking the drug to a patient using steroids during the lactation period.
I3. At drug level	Recommendation for drug change in accordance with kidney functions.
I4. Other	Recommendation for referral to the relevant branch after discharge for uncontrolled chronic disease
Intervention Acceptance	
A1. Intervention accepted	Recommendation to discontinue drug use in patients using two different antiemetics
A2. Intervention not accepted	Recommendation for analgesic change for the patient with intense pain.
A3. Other	It was suggested that the patient with tinnitus was told that the problem could be caused by medication, and referral to the relevant branch physician was recommended.
Status of the DRP	
O0. Problem status unknown	The patient who needed LMWH could not be followed up due to discharge to a different service.
O1. Problem solved	The dose of meropenem was adjusted according to the patients’ need.
O2. Problem partially solved	The patient's electrolyte deficiency was associated with chemotherapy.
O3. Problem not solved	The patient who needed dose reduction was referred to the relevant branch for dose correction after discharge.

## Discussion

Chest diseases can occur due to different reasons. In particular, the presence of diseases increases mortality and morbidity increase the need for health care services. The most common DRPs were mainly drug (C1, 49.37%) or dose selection (C3, 40.51%). The intervention made by CPs for DRPs were mainly at prescriber level (I1, 88.16%) and the acceptance rate of the intervention was (A1, 84.21%) (table 3 and 4). Similar to literature patients with higher number of comorbidities, polypharmacy, increased Charlson Comorbidity Index (CCI) scores, previous history of frequent hospital admission and patient with infectious diseases were more prone to DRPs and pDDIs (15–17).

Most of the patients included in our study were hospitalized due to pneumonia. In terms of its high incidence, increased mortality and morbidity values, pneumonia creates the need for high quality health care. It has been shown in the literature that clinical pharmacy services led to beneficial results in patients with pneumonia. Drug-related problems can cause serious problems in patients with pneumonia from different perspectives. In a study conducted by Bekele et al., reported that the rate DRPs in patients who were hospitalized due to infectious diseases was reported as 71.51%. The irrational selection of ceftriaxone, and this omission was constituting 44.7% of existing DRPs (Bekele et al., 2021). In another study conducted in Spain, nearly half (45.1%) of hospitalized individuals with pneumonia were reported to have DRPs (Garin et al., 2021).

As stated in the literature, similar outcomes were obtained in our study. A statistically significant difference was found between DRPs detected in the CG and IG. Consistent with other studies in the literature, the active participation of the clinical pharmacist in the healthcare team has ensured the prevention of possible or existing DRPs (Daneman et al., 2013; Roberts et al., 2014; Ngocho et al., 2020; Oliver et al., 2021). The results we obtained in our study reinforce the role of the clinical pharmacist in the detection and prevention of DRPs. In addition, many different tools and methods such as antibiotic management programs, biomarkers, and Computerized physician order entries (CPOE) must be applied to ensure the effectiveness and safety of compliance with the guidelines. Computerized physician order entries (CPOE) and clinical decision support systems (CDSSs) are other technological tools that can be used in interventions to prevent adverse drug events (ADRs). Although these sophisticated systems are effective in reducing DRPs, they cannot override the role of the clinical pharmacist in preventing DRPs (Daneman et al., 2013).

One of the most prevalent DRPs in patients with pneumonia, particularly community-acquired pneumonia (CAP), is irrational antibiotic selection. In a research study conducted in community pharmacies, it was discovered that inappropriate antibiotic selection affects both adults (57.7%) and children (56.6%) in antibiotic prescriptions gathered from 22 pharmacies (Dorj et al., 2013). In a study conducted by Wattengel et al. (2019) with participation of 518 outpatients with CAP reported that 69% of the patients used an inappropriate antibiotic. On the other hand, inappropriate medication was prescribed in 76.7% of patients due to their comorbidities (Wattengel et al., 2019). In another study conducted in Thailand, it was reported that 52% of patients with severe CAP used antibiotic regimens that did not adhere with the IDSA/ATS guidelines (Wongsurakiat and Chitwarakorn, 2019). Outpatients should be treated with beta-lactam, macrolide, or tetracycline group antibiotics, in accordance with the current guidelines for the management of community-acquired pneumonia (Bradley et al., 2011; Gralnek et al., 2015). In a retrospective study investigating the use of fluoroquinolone antibiotics, it was shown that 71% of the prescribed fluoroquinolones were over-prescribed for low-risk patients (Thiessen et al., 2017). In another retrospective study involving 156 adult patients diagnosed with CAP in Canada, it was reported that doctors prescribed fluoroquinolones for 80.8% of their patients, even though it was not needed for the patients (Yu et al., 2020). The unnecessary use of fluoroquinolones increases the risk of side effects such as tendon rupture, tendinitis, and aortic rupture and may contribute to the development of antibiotic resistance (Bethesda, 2012; Stephenson et al., 2013; Pasternak et al., 2018). In a retrospective cohort study involving a clinical pharmacist, it was reported that the use of fluoroquinolones in CAP decreased by implementation of clinical pharmacy services to practice (Kulwicki et al., 2019).

In our study, it was observed that both chest disease specialists and other physicians involved in the treatment comply with the current diagnosis and treatment guidelines. In addition, obtaining the approval of the infectious disease specialist before antibiotic selection contributed to the minimum level of problems related to antibiotic selection in our case. Although problems related to antibiotic selection were observed at a minimum level in our study, it should be kept in mind that this situation was observed in a university hospital that provides the tertiary level of healthcare. As it is often stated in the literature, the irrational use of antibiotics can occur in different healthcare units. For all reasons, the clinical pharmacist has a vital task in terms of appropriate drug selection. Therefore, the role of the clinical pharmacist in antibiotic management programs should be assessed.

One of the most frequent chest diseases is chronic obstructive lung diseases which contributes to morbidity, mortality, and health care costs. The patient with COPD requires increased healthcare services and COPD related hospital admissions have quite complex and usually requires longer hospitalization periods. There are several risk factors identified which contribute to increased readmission rate such as lack of follow-up and decreased adherence to pharmacologic guidelines (Press et al., 2019). According to the Cherian et al. study pharmacist intervention has significant effects on COPD related readmission. In addition to reduced readmission rates pharmacist interventions resulted as decreased the hospital stay, escalation of COPD therapy, increased pulmonology, physical therapy, and palliative consults in Cherian et al. results (Cherian et al., 2003). Similar to Cherian et al. results, CP interventions reduced the 30-day readmissions in current investigations of our study. Involvement of clinical pharmacist allowed increased for therapy optimization and decreased potential or present DRPs in favor of patients.

Another frequently encountered problem in hospitalized patients is the need for dose adjustments for prescribed antibiotics. According to a Canadian trial on pneumonia, administered antibiotic dosages were often greater than needed (Yu et al., 2020). It should be kept in mind that such irrational use of antibiotics would increase antibiotic resistance. In addition to all these precautions, critically ill patients in CAP therapy require individualized dosing based on disease severity, established pathogen susceptibility tests, and infection-causing bacteria.

Among the interventions made within the scope of our study, the dose adjustments of the prescribed drugs were noted as a frequent intervention by the clinical pharmacist. It is a known fact that individuals with chronic diseases such as kidney failure, Type 2 DM, and hypertension, especially together with pneumonia, COPD or any other chest disease requires dose adjustments (Apikoglu-Rabus et al., 2016; Rankin et al., 2018). Antibiotics, anticoagulant drugs, antiadrenergic and anticholinergic drugs are among the drugs whose dosage is adjusted by the clinical pharmacist (Ayhan et al., 2022).

It is available in the literature that comorbidities in hospitalizations have a direct effect on mortality, morbidity, and readmission. Similarly, comorbidities were associated with mortality in pneumonia patients (Wesemann et al., 2015). The increasing number of comorbidities directly affects the increase in the number of drugs used. The simultaneous use of more than one drug may cause increased potential drug–drug interactions (pDDIs) and DRPs (Gamble et al., 2014; Gülçebi İdriz Oğlu et al., 2016; Noor et al., 2019). A study held by Noor et al. (2019) on a population using more than 10 drugs reported that 73.1% of the participants had at least one pDDI. On the other hand, according to the results of Gamble et al. (2014)’s study, polypharmacy was reported in 75% of patients with CAP. Pneumonia may trigger other comorbid diseases and cause an impact on the incidence of events such as acute myocardial infarction, heart failure, stroke, venous thromboembolism, and cancer (Yousufuddin et al., 2018). Consideration of the link between pneumonia and comorbidities will assist in the identification of high-risk individuals. While there is no particular guideline for multimorbidities currently, improved outcomes can be attained *via* careful monitoring of patients during hospitalization and long-term follow-up.

High comorbidity numbers in our sample are noteworthy in both the CG and IG (mean number of comorbidities per patient is 3.71 ± 1.74 and 3.66 ± 1.67, respectively). This seems to be related to the number of drugs directly affected by the increased number of comorbidities. Clinical pharmacy services offer important solution alternatives for treatment optimization in individuals with complex drug regimens. In light of the current literature and the results we have obtained, it can be predicted that adding clinical pharmacy services to the routine workflow in the pulmonology service will contribute to the success of treatment.

Readmission is among the frequently encountered problems for the patients admitted to chest disease wards. Readmissions after discharge directly affect mortality and morbidity, as well as reduce health-related quality of life and reduce the capacity of the health system (Press et al., 2019). Similarly, risk factors for hospital readmission in lower respiratory tract infections are among the most important problems to be considered. Possible risk factors for hospital readmission in patients admitted to chest disease ward include male gender, age 70 or older, longer hospital stay, and a multi-comorbidity score greater than 10 (Press et al., 2019; Mannino and Thomashow, 2015; Faverio et al., 2020). A standard treatment protocol has not been adopted for hospital readmission in CAP patients. Frequently used inhaler drugs can show different profiles in different comorbidities. Inhaled steroids may be useful in CAP in patients with COPD, but anticholinergics could beneficially option asthma patient with pneumonia. The effect of inhaled medication on the risk of CAP is difficult to separate from the effect of COPD or asthma severity. These relationships, on the other hand, may not be causative; nonetheless, they may draw attention to the significance of inhaled treatment in COPD and asthma patients. (Faverio et al., 2020). In pediatric patients infected with Mycoplasma pneumonia, hospital readmission within 90 days of discharge was associated with age, fever, and influenza-A coinfection at the time of hospitalization (Wang et al., 2019). However, risk factors for hospital readmission within 30 days in adult patients include age, previous hospitalizations, respiratory diseases, heart failure, liver disease, and a lack of health care at home (Toledo et al., 2018).

The effects of clinical pharmacy services that were investigated by Lisenby et al. showed that patients who underwent drug reconciliation, drug review, pre-discharge patient education, and post-discharge services by the clinical pharmacist were associated with a reduced rate of 30-day readmission, and CP provided more than 200 interventions (Lisenby et al., 2015). These data reveal descriptive information about the necessity of the pharmaceutical care service provided by the clinical pharmacist. Pharmaceutical care services offered by CP to patients in the high-risk group can increase the quality of healthcare, decrease economic output, and increase the level of welfare (Lisenby et al., 2015). According to Jang and Ahn (2020), conditions such as chronic lung disease, chronic kidney disease, treatment failure, and decompensation of comorbidities have been associated with hospital readmission within 30 days of discharge unrelated to pneumonia.

Regarding the discharge counseling, a single-center pilot study focused on the impact of pharmacists on care transitions and readmission rates using interventions such as drug therapy consensus, therapeutic recommendations, discharge instructions, and follow-up. After pharmacist intervention readmission rate reduced. Changing the route of drug administration was the most frequent intervention after optimizing therapy in the study (Faverio et al., 2020). Similar to literature CP interventions of current study were mainly focused on drug and dose selection. In addition to CPs‘ intervention during the hospital stay discharge consultations and patient education would have beneficial effects on the decreased readmission rate.

### Limitations of the study

Although the results presented within the scope of our research showed the possible benefits of clinical pharmacy services in the chest diseases service, our study has some limitations. The first thing that stands out among these limitations is that the study was conducted in a single center and suggestion made by a single clinical pharmacist. This situation may have caused the formation of contamination bias in our study. The limited generalizability of the results due to the sampling from a single center is another important point to be considered in future studies. Also, majority of the participants was consisted of geriatric patients this situation could affect generalizability of the study. Another limitation of our study is that the randomization was performed by an only one group of researchers. Another noteworthy suggestion is to increase the number of patients in future studies.

## Conclusion

According to our results, the clinical pharmacist intervention can lead to positive outcomes in assessing, preventing, and solving the DRPs, especially in individuals with multiple diseases, the probability of DRPs increases and the success of healthcare may be negatively affected. In light of the current evaluations in the literature and the data we obtained, it can be said that the services provided by clinical pharmacists have the potential to increase the quality of health.

As demonstrated by different researchers in the literature, clinical pharmacy services were found to be effective in reducing hospitalizations after discharge as in our study. In the analyses made between the control and intervention groups, 30-day readmission was statistically significantly decreased in patients who received clinical pharmacy services in addition to standard care.

Based on the data we obtained in our study and the studies in the literature, we think that clinical pharmacists providing cognitive pharmacy services in cooperation with the healthcare team in the hospital environment will contribute significantly to the success of treatment.

## Data Availability

The raw data supporting the conclusion of this article will be made available by the authors, without undue reservation.
